# Seroprevalence and distribution of bovine and porcine cysticercosis in rural areas of Mpumalanga province, South Africa

**DOI:** 10.4102/ojvr.v93i1.2261

**Published:** 2026-04-30

**Authors:** Usman A. Ahmed, Malitaba A. Mlangeni, Ana M. Tsotetsi-Khambule

**Affiliations:** 1 Department of Life and Consumer Sciences, College of Agriculture and Environmental Sciences, University of South Africa, Johannesburg, South Africa

**Keywords:** cysticercosis, Taenia spp., seroprevalence, cattle, pigs, zoonosis, ELISA

## Abstract

Cysticercosis is a neglected zoonotic disease caused by the larval stages of *Taenia asiatica, T. saginata* and *T. solium*. Its prevalence varies across regions, with limited data available in South Africa, particularly in Mpumalanga province. This study aimed to determine the seroprevalence and distribution of bovine and porcine cysticercosis in rural communities of Mpumalanga province, South Africa. A cross-sectional survey was conducted from 2021 to 2022 in Ehlanzeni and Nkangala districts. Blood samples were collected from 384 cattle and 336 pigs and analysed using the Apdia^®^ cysticercosis AgELISA kit. Logistic regression was used to estimate odds of infection, with statistical significance set at *p* < 0.05. Overall seroprevalence of bovine cysticercosis was 35.2% (95% confidence interval [CI]: 30.5–40.2), while porcine cysticercosis was 4.8% (95% CI: 2.8–7.6). Seroprevalence varied across districts and municipalities in Mpumalanga province, with evidence of spatial heterogeneity in both cattle and pigs. The higher prevalence in cattle is likely because of free-range grazing practices, increasing exposure to Taenia-contaminated environments. Pigs, mostly penned, had lower infection rates.

**Contribution:**

This study provides the first baseline data on bovine and porcine cysticercosis in Mpumalanga province, highlighting the need for improved livestock management, sanitation, farmer education and targeted surveillance to reduce transmission.

## Introduction

Cysticercosis is a parasitic zoonotic disease caused by the larval stages of Taenia species, notably *Taenia saginata* in cattle and *Taenia solium* in pigs (Assana et al. 2013; Dorny et al. 2003). The disease has significant public health and economic implications, particularly in low- and middle-income countries where livestock production systems are closely integrated with human settlements (Assana et al. 2013; Nkouawa et al. 2017; World Health Organization [WHO] & Food and Agriculture Organization [FAO] 2005). In food-producing animals, cysticercosis results in carcass condemnation, reduced market value and trade restrictions, while in humans, *T. solium* infection may lead to taeniasis and neurocysticercosis, a major cause of preventable epilepsy worldwide (Nkouawa et al. 2017; Veary & Manoto 2008; WHO & FAO 2005).

In sub-Saharan Africa, cysticercosis remains endemic, especially in rural communities characterised by poor sanitation, free-ranging livestock production systems and limited access to veterinary and meat inspection services (Assana et al. 2013; Krecek et al. 2008; Tsotetsi-Khambule et al. 2018). South Africa is not exempt from this burden, with previous studies reporting variable prevalence of bovine and porcine cysticercosis across different provinces (Dube et al. 2024; Sithole et al. 2018; Tsotetsi-Khambule et al. 2017). However, data from rural areas of Mpumalanga province remain limited, despite the province’s substantial livestock population and its proximity to international borders that may influence livestock movement and disease transmission (Mpumalanga Provincial Government 20; Nkosi, Bekker & Hoffman 2019). Generating updated epidemiological data from such settings is therefore essential for informing targeted surveillance and control strategies. Diagnosis of cysticercosis in livestock has traditionally relied on post-mortem meat inspection, which is regarded as the conventional reference method (Joshi et al. 2019; WHO & FAO 2005). However, several studies have demonstrated that routine meat inspection has low sensitivity, particularly for detecting light or early infections, and may substantially underestimate the true prevalence of infection (Laranjo-González et al. 2016; Sithole et al. 2018). Serological methods, such as antigen-detection enzyme-linked immunosorbent assay (ELISA), have been shown to provide higher sensitivity for detecting active infections in live animals (Dorny et al. 2003; Eichenberger et al. 2013; Harrison et al. 1989). These assays facilitate large-scale epidemiological surveys and are particularly suitable for field-based studies in rural settings where access to formal abattoirs is limited (Dorny et al. 2010; Tsotetsi-Khambule et al. 2017). Consequently, ELISA-based approaches are increasingly recommended for surveillance and prevalence studies of Taenia spp. infections in endemic areas (Dorny et al. 2003; WHO & FAO 2005).

The present study aimed to determine the seroprevalence and spatial distribution of bovine and porcine cysticercosis in selected rural areas of the Ehlanzeni and Nkangala districts of Mpumalanga province, South Africa, using antigen-detection ELISA. Specifically, the study sought to estimate the prevalence of cysticercosis in cattle and pigs and to describe the distribution of infection across selected municipalities within the study area, thereby contributing to improve understanding of cysticercosis epidemiology and supporting evidence-based interventions within a One Health framework.

## Research methods and design

### Study area

The study was conducted in Ehlanzeni and Nkangala districts of Mpumalanga province, South Africa. Mpumalanga province is located in the north-eastern region of the country, bordering Mozambique and Eswatini, and lies approximately between latitudes 24°30’S and 27°00’S and longitudes 30°30’E and 32°00’E.

**FIGURE 1:**
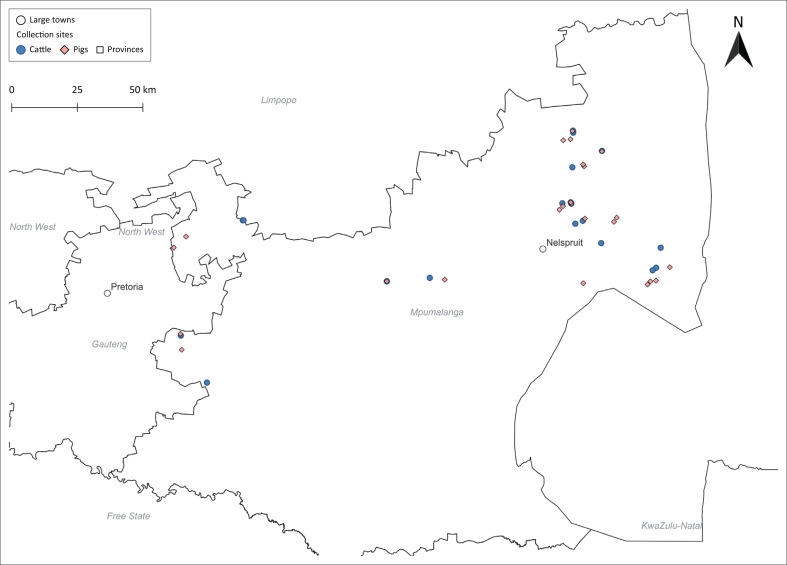
Geographic distribution of collection sites for cattle and pigs in Mpumalanga, South Africa.

The province experiences a predominantly subtropical climate, with warmer conditions in the Lowveld and cooler temperatures in the Highveld. Mean annual temperatures generally range from approximately 18 °C to 28 °C, while average annual rainfall ranges between 500 mm and 1000 mm, with most rainfall occurring during the summer months. Livestock production in the study districts is mainly extensive, dominated by smallholder cattle and pig farming systems. The geographical location of the study area is shown in Figure 1.

### Study design

A cross-sectional study design was employed to determine the seroprevalence and spatial distribution of bovine and porcine cysticercosis in selected rural areas of Mpumalanga province. Sampling was conducted at a single point in time across selected municipalities within Ehlanzeni and Nkangala districts.

### Sample size

The sample size was calculated using a standard single-proportion prevalence formula as described by Thrusfield (2005):


$$n=\frac{1.96^{2}\times P_{\exp}\times (1-P_{\exp})}{d^{2}}$$ [Eqn 1]

where

*n* = required sample size,

*P*_exp_ = expected seroprevalence (expressed as a proportion),

*d* = absolute precision.

In the absence of prior prevalence data for cysticercosis in Mpumalanga province, an expected prevalence of 50% was assumed to maximise sample size, with a 95% confidence level and a 5% margin of error. This yielded a minimum required sample size of 384 animals per species.

To account for potential non-response and sample loss, additional samples were targeted where feasible. Ultimately, a total of 384 cattle and 336 pigs were included in the analysis, as pig availability was limited in some rural households.

### Sample collection and processing

Blood samples were collected from September 2021 to November 2022 with the assistance of registered Mpumalanga Animal Health Technicians (AHT). A volume of 1.5-inch needle for porcine samples is drawn into anticoagulant-free vacutainers. Samples were immediately placed in cooler boxes with ice gel packs, maintained at a temperature of 4 °C, and transported to the laboratory located at the University of South Africa (UNISA) Florida campus in South Africa.

Blood samples were allowed to clot and then centrifuged at 15 000 revolutions per minute (rpm) for 10 min to separate the serum from the cellular fraction. The clear supernatant (serum) was carefully aspirated, and a 1.5 mL aliquot was transferred into Eppendorf^®^ plastic microtubes. Each microtube was meticulously labelled with unique identifiers, including sample and farm numbers, to maintain precise tracking. The labelled serum aliquots were stored at −20 °C until required for subsequent analytical procedures.

### Laboratory analysis

Detection of circulating Taenia spp. antigens was performed using a commercial antigen-detection enzyme-linked immunosorbent assay (ELISA) kit (Apdia^®^ Cysticercosis AgELISA; Apdia nv, Turnhout, Belgium), following the manufacturer’s instructions. Briefly, serum samples were pre-treated with trichloroacetic acid (TCA) solution, centrifuged and neutralised with buffer. Pre-treated samples were added in duplicate to 96-well microplates and incubated at 37 °C for 15 min with shaking. After washing, conjugate solution was added and incubated, followed by the addition of chromogen solution and incubation at room temperature. The enzymatic reaction was stopped using the stop solution, and optical density (OD) was measured at 450 nm using an ELISA plate reader (Varioskan™ 3001 model, manufactured by Thermo Electron Corporation, in Waltham, Massachusetts, United States) within 15 min.

Samples were classified as positive or negative based on the cut-off values provided by the manufacturer:

- Cut-off and antigen index calculation

The cut-off value was calculated as follows:

- Cut-off = Mean OD of negative controls × 3.5

The antigen index (Ag Index) for each sample was calculated using the formula:

- Ag Index = Mean OD of sample / Cut-off

### Justification for the use of antigen enzyme-linked immunosorbent assay and limited post-mortem examination

Although post-mortem meat inspection is traditionally regarded as the gold standard for the diagnosis of cysticercosis, its application in this study was limited because of several contextual factors. In the study areas, livestock slaughter is predominantly informal and often occurs outside registered abattoirs, limiting access to systematic post-mortem examination. In addition, routine meat inspection has been shown to have low sensitivity, particularly for detecting light or early infections.

Antigen-detection ELISA was therefore selected as a more sensitive and practical tool for epidemiological surveillance in live animals, allowing detection of active infections under field conditions and enabling broader population-level assessment without the ethical and logistical constraints associated with slaughter-based diagnostics.

### Statistical analysis

Data were captured in Microsoft Excel 2010 sheets and subsequently analysed using IBM Statistical Package for the Social Sciences (SPSS) Statistics version 30.0.0.0 (172) (IBM Corp., Armonk, New York, United States). The outcome variable was serological status as determined by antigen-detection ELISA and classified as positive or negative.

Descriptive statistics were used to calculate overall and stratified seroprevalence estimates for bovine and porcine cysticercosis, expressed as percentages with corresponding 95% confidence intervals (CI). Univariable logistic regression analysis was initially performed to assess associations between ELISA sero-positivity and explanatory variables, including animal species (cattle or pigs), district (Ehlanzeni or Nkangala) and municipality. Variables with a *p*-value ≤ 0.20 in the univariable analysis were considered for inclusion in multivariable logistic regression models. Adjusted odds ratios (ORs) with 95% CIs were calculated, and statistical significance was set at *p* < 0.05.

### Ethical considerations

The study received ethical clearance from the Animal Research Ethics Committee, (CAES_AREC) of the University of South Africa with reference number (2021/CAES_AREC/191), and an approval under Section 20 of the *Animal Diseases Act of 1984* granted by the Department of Agriculture, Land Reform and Rural Development (DALRRD) to collect samples in Mpumalanga province. Informed consent was obtained from each animal owner before the collection of data and samples from cattle and pigs in Mpumalanga province.

## Results

A total of 384 cattle and 336 pigs were sampled from Ehlanzeni and Nkangala districts of Mpumalanga province. In Nkangala District, 192 cattle and 146 pigs were sampled, while in Ehlanzeni District, 192 cattle and 190 pigs were sampled. The initially targeted porcine sample size of 384 could not be achieved because of limited availability of pigs in some rural households.

### Seroprevalence of bovine cysticercosis

The overall seroprevalence of bovine cysticercosis in Mpumalanga province was 35.2% (*n* = 135/384). A higher prevalence was observed in Ehlanzeni District (38.5%; *n* = 74/192) compared to Nkangala District (31.8%; *n* = 61/192), although this difference was not statistically significant (*p* = 0.165).

In Nkangala District, bovine cysticercosis prevalence varied markedly across municipalities (Table 1). Thembisile Hani Municipality recorded the highest seroprevalence at 65.6% (*n* = 42/64; OR = 16.95; 95% CI: 6.47–44.39; *p* < 0.001), followed by Victor Khanye at 18.8% (*n* = 12/64; OR = 2.75; 95% CI: 1.00–7.54; *p* = 0.05). Emakhazeni Municipality recorded the lowest prevalence at 10.9% (*n* = 7/64) and served as the reference category.

**TABLE 1:** Seroprevalence of cysticercosis in cattle in Nkangala district, Mpumalanga province.

Municipality	Total samples	Positive samples	Prevalence (%)	Odds ratio (OR)	95% CI	*p*-value
Emakhazeni	64	7	10.9	-	-	-
Thembisile Hani	64	42	65.6	16.95	6.47–44.39	< 0.001
Victor Khanye	64	12	18.8	2.75	1.00–7.54	0.050

**Overall**	**192**	**61**	**31.8**	-	-	-

CI, confidence interval.

In Ehlanzeni District, Bushbuckridge Municipality exhibited the highest bovine cysticercosis seroprevalence at 53.1% (*n* = 34/64). Compared with Bushbuckridge, significantly lower prevalence was observed in Nkomazi (26.6%; *n* = 17/64; OR = 0.33; 95% CI: 0.16–0.66; *p* = 0.002) and Mbombela (35.9%; *n* = 23/64; OR = 0.49; 95% CI: 0.25–0.95; *p* = 0.04) (Table 3).

### Seroprevalence of porcine cysticercosis

The overall seroprevalence of porcine cysticercosis in Mpumalanga province was 4.8% (*n* = 16/336). No statistically significant difference was observed between Nkangala District (4.8%; *n* = 7/146) and Ehlanzeni District (4.7%; *n* = 9/190) (*p* = 1.000).

In Nkangala District, porcine cysticercosis prevalence showed limited variation across municipalities (Table 2). Thembisile Hani Municipality recorded a prevalence of 6.3% (*n* = 4/64; OR = 1.36), Emakhazeni recorded 4.7% (*n* = 3/63), while no sero-positive cases were detected in Victor Khanye Municipality (*n* = 0/19). Because of the low number of positive cases, differences between municipalities were not statistically significant.

**TABLE 2:** Seroprevalence of cysticercosis in pig in Nkangala district, Mpumalanga province.

Municipality	Total samples	Positive samples	Prevalence (%)	Odds ratio (OR)	95% CI	*p*-value
Emakhazeni	63	3	4.7	-	-	-
Thembisile Hani	64	4	6.3	1.36	6.47–44.39	< 0.001
Victor Khanye	19	0	0.0	-	1.00–7.54	0.050

**Overall**	**146**	**7**	**4.8**	-	-	-

Note: Odds ratios and confidence intervals were not estimated for municipalities with zero positive cases.

CI, confidence interval.

In Ehlanzeni District, Nkomazi Municipality recorded the highest porcine cysticercosis seroprevalence at 12.9% (*n* = 8/62; OR = 8.00; 95% CI: 0.98–65.23; *p* = 0.05), followed by Bushbuckridge at 15.6% (*n* = 10/64). No sero-positive cases were detected in Mbombela Municipality (*n* = 0/64) (Table 4).

**TABLE 3:** Seroprevalence of cysticercosis in cattle in Ehlanzeni district, Mpumalanga province.

Municipality	Total samples	Positive samples	Prevalence (%)	Odds ratio (OR)	95% CI	*p*-value
Bushbuckridge	64	34	53.1	-	-	-
Nkomazi	64	17	26.6	0.33	0.16–0.66	0.002
Mbombela	64	23	35.9	0.49	0.25–0.95	0.040

**Overall**	**192**	**74**	**38.5**	-	-	-

CI, confidence interval.

**TABLE 4:** Seroprevalence of cysticercosis in pigs in Ehlanzeni district, Mpumalanga province.

Municipality	Total samples	Positive samples	Prevalence (%)	Odds ratio (OR)	95% CI	*p*-value
Bushbuckridge	64	10	15.6	-	-	-
Nkomazi	62	8	12.9	8.00	0.98–65.23	0.05
Mbombela	64	0	0.0	-	0.25–0.95	0.04

**Overall**	**190**	**9**	**4.7**	-	-	-

CI, confidence interval.

### Factors associated with cysticercosis sero-positivity

Univariable logistic regression analysis was conducted to assess associations between cysticercosis sero-positivity and animal species, district and municipality. However, sparse data and zero-cell counts in several municipalities limited the stability of multivariable logistic regression models. Consequently, results are presented primarily as prevalence estimates with corresponding ORs and 95% CIs derived from univariable analyses.

## Discussion

The overall seroprevalence of bovine cysticercosis in Mpumalanga province was 35.2% (*n* = 135/384; 95% CI: 30.5–40.2), while the seroprevalence of porcine cysticercosis was 4.8% (*n* = 16/336; 95% CI: 2.8–7.6). These findings confirm that cysticercosis remains endemic in rural areas of Mpumalanga province and demonstrate marked spatial heterogeneity across districts and municipalities. Similar prevalence patterns have been reported in other endemic regions of South Africa and sub-Saharan Africa, where extensive livestock production systems and inadequate sanitation contribute to sustained transmission of Taenia spp. infections (Dube et al. 2024; Krecek et al. 2008; Sithole et al. 2018; Tsotetsi-Khambule et al. 2017).

The higher seroprevalence observed in cattle compared with pigs is consistent with previous studies reporting greater exposure of cattle to contaminated grazing environments, particularly in communal farming systems (Assana et al. 2013; Laranjo-González et al. 2016; Tsotetsi-Khambule et al. 2018). The use of antigen-detection ELISA in the present study enabled detection of active infections in live animals, thereby providing more sensitive prevalence estimates than routine post-mortem meat inspection, which has been shown to underestimate infection because of its low sensitivity, especially in light or early infections (Dorny et al. 2003; Eichenberger et al. 2013; Harrison et al. 1989; Joshi et al. 2019; WHO & FAO 2005).

This study had several limitations. Data on animal management practices and husbandry systems were not collected and could therefore not be analysed as potential risk factors for cysticercosis sero-positivity, as has been demonstrated in other endemic settings (Assana et al. 2013; Krecek et al. 2008; Tsotetsi-Khambule et al. 2018). In addition, age stratification of pigs was not recorded during sampling, limiting assessment of age-related infection risk, which has been shown to influence cysticercosis prevalence in previous studies (Dorny et al. 2003; Sithole et al. 2018). Human taeniasis data were also unavailable for the study area, which restricted direct evaluation of zoonotic transmission risk, despite the well-documented association between porcine cysticercosis and human taeniasis and neurocysticercosis (Nkouawa et al. 2017; Veary & Manoto 2008; WHO & FAO 2005). Despite these limitations, the use of antigen-detection ELISA allowed for reliable estimation of active infections and provided valuable epidemiological insight into the spatial distribution of cysticercosis in rural Mpumalanga province, consistent with findings from similar field-based studies (Dorny et al. 2003; Eichenberger et al. 2013; Harrison et al. 1989).

## Conclusion

Bovine and porcine cysticercosis remain present in rural areas of Mpumalanga province, with clear spatial variation across districts and municipalities. The application of antigen-detection ELISA provided valuable epidemiological data that may be underestimated by routine meat inspection alone (Dube et al. 2024; Eichenberger et al. 2013; Harrison et al. 1989; Joshi et al. 2019). These findings underscore the need for strengthened surveillance systems and integrated control strategies informed by a One Health approach (Nkouawa et al. 2017; Nsadha et al. 2014). Further research incorporating livestock management practices and human infection data is recommended to guide effective prevention and control measures in endemic communities (Marshalla et al. 2016; Ngowi et al. 2019).
